# Anticancer Effect of Puerarin on Ovarian Cancer Progression Contributes to the Tumor Suppressor Gene Expression and Gut Microbiota Modulation

**DOI:** 10.1155/2022/4472509

**Published:** 2022-07-28

**Authors:** Yongju Ye, Yang Gao, Yuan Fang, Lixia Xu, Fule He

**Affiliations:** ^1^Department of Gynaecology, Lishui Hospital of Traditional Chinese Medicine, Lishui City, 323000 Zhejiang Province, China; ^2^Clinical laboratory, Lishui Hospital of Traditional Chinese Medicine, Lishui City, 323000 Zhejiang Province, China; ^3^Zhejiang Chinese Medicine Museum, Zhejiang Chinese Medical University, Hangzhou City, 310053 Zhejiang Province, China

## Abstract

Ovarian cancer (OC) causes more deaths than any other cancer of the female reproductive system due to its late presentation and malignant nature. Although significant progress has been made in the diagnosis and treatment of OC over the last decade, chemotherapeutic drug resistance and cancer recurrence remain serious challenges in OC management. In the field of cancer therapy, traditional Chinese herbal medicines and their active compounds have been widely reported to have favorable therapeutic effects on cancer. Recent studies have also revealed the protective effect of puerarin in cancer, but the exact role and underlying mechanism of puerarin in OC remain unclear. Here, we established *in vivo* and *in vitro* OC models to evaluate the anticancer effect of puerarin. It was found that puerarin significantly inhibited OC cell viability and proliferation and induced cell apoptosis. In OC model mice, puerarin treatment suppressed tumor formation and modulated the gut microbiome. In addition, the expression of tumor suppressor genes was activated by puerarin *in vitro* and *in vivo*. These findings add to the existing knowledge on the usefulness of herbal active ingredients for the prevention and treatment of OC and provide a new perspective regarding the therapeutic potential of puerarin in cancer.

## 1. Introduction

Although ovarian cancer (OC) accounts for only 2.5% of all malignancies among women, it accounts for 5% of all cancer deaths due to its relatively high mortality rate [1]. Based on the report of Global Cancer Statistics, there were approximately 314,000 newly diagnosed cases of OC and 207,200 new deaths in 2020 worldwide [2]. About 90-95% of the cases were primary carcinomas of the ovary, with the most common being epithelial serous carcinoma. Currently, OC screening is not widely recommended for the general female population at average risk; even for women at increased risk, screening results in about 4 out of 5 OC patients being diagnosed with advanced disease [3]. In the USA, most serous carcinomas of OC are diagnosed at stage III (51%) or IV (29%), for which the 5-year cause-specific survival rates of patients diagnosed in 2007 through 2013 were 42% and 26%, respectively [4]. The diagnosis and treatment of OC have improved significantly over the last decade, with a flurry of optimized surgical strategies, newly identified molecular markers, and drug approvals [5, 6]. Nevertheless, new opportunities come with new challenges, and chemotherapy drug resistance and cancer recurrence remain serious challenges in OC management. Due to its clinical, biological, and molecular complexity, OC is still considered one of the most difficult tumors to cure [7].

In the field of cancer diagnosis and therapy, the tumor suppressor genes and the corresponding tumor promoter genes (named oncogenes) have attracted much attention. *P53* is one of the well-known tumor suppressor genes, and its wild type can suppress tumor progression via multiple pathways, while its mutation can promote cancer cell evasion and aggravate tumor progression [8]. *PTEN* is another potent tumor suppressor that can inhibit tumor progression by modulating a variety of biological processes, such as cell survival, proliferation, and metabolism [9]. The loss of *PTEN* has been reported as a frequent driver in OC and has been distinctly associated with the levels of hormonal receptors, including the estrogen receptor, progesterone receptor, and androgen receptor, as well as the percentage of CD8+ tumor-infiltrating lymphocytes in high-grade serous and clear cell histotypes of OC [10]. Therefore, candidate agents targeting *P53* and *PTEN* to inhibit the mutations of these genes have shown potential applicability in inhibiting the growth and metastasis of OC and are worth exploring for improving OC therapy [11]. Studies have also shown that the tumor suppression activity of these genes demonstrates a correlation with the tumor microenvironment and immunosuppression in cancer, including OC [12, 13].

Traditional Chinese herbal medicines and their active compounds have been proven to have a unique therapeutic effect on various cancers, along with low toxicity and synergistic effects [14]. Puerarin is an isoflavone derivative isolated from the traditional Chinese herbal medicine *Pueraria lobata* (Willd.) Ohwi. It has been widely used in the treatment of cardiovascular and cerebrovascular diseases, diabetes, and diabetic complications due to its wide spectrum of pharmacological properties, such as the capacity to lower blood pressure, reduce myocardial oxygen consumption, expand coronary vessels, and control blood sugar [15]. In addition to the significant role in cardiovascular and cerebrovascular diseases, recent studies also revealed the protective potential of puerarin in cancer. The anticancer mechanism involves the regulation of the Bcl-2 proteins, caspase family, NF-*κ*B signaling pathway, PI3K/AKT/mTOR signaling pathway, and JNK/ESR1/2 signaling pathway, among others [16]. Duan et al. found that puerarin can target SIRT1 to induce cell apoptosis in platinum-resistant OC cells [17]. However, the exact role and underlying mechanism of puerarin in OC remain elusive.

Hence, based on previous reports on the protective potential of puerarin in OC, we hypothesized that puerarin might target the tumor suppressor genes and modulate the gut microbiota to stimulate the tumor microenvironment, thus inhibiting OC progression. In this study, the anti-OC effect of puerarin was studied in SKOV3 and NuTu-19 cells. Furthermore, an OC rat model was established, and the antitumor effect of puerarin and its underlying mechanism were explored. This study hopes to provide a basic reference for the application of puerarin in OC therapy.

## 2. Materials and Methods

### 2.1. Chemicals, Antibodies, and Kits

Puerarin was purchased from Macklin (Art. No. P816259, China) with purity ≥ 98% (HPLC). Antibodies against P53, P21, PTEN, BRCA1, BIRC5, CTGF, Bax, Bcl-2, caspase 3, cleaved caspase 3, GAPDH, Ki67, and PCNA were purchased from Affinity Biosciences (USA); IgG H&L (Alexa Fluor® 488) was from Abcam (UK); CD3, CD4, and CD8 were from BD Biosciences Pharmingen (USA). The CCK-8 kit was obtained from MedChemExpress (USA); the cell apoptosis kit was obtained from BD Biosciences Pharmingen (USA); the EdU-594 cell proliferation detection kit was obtained from Beyotime Biotechnology (China); the TUNEL staining kit was obtained from Servicebio (China); ELISA kits for IL-6 and TNF-*α* were purchased from MEIMIAN (China); the ELISA kit for CA125 was purchased from ZSGB-BIO (China).

### 2.2. Cell Culture and Grouping

Human OC cell line SKOV3 and Fischer 344 rat OC cell line NuTu-19 were purchased from iCell Bioscience Inc. (China). Cells were cultured in the RPMI 1640 medium (HyClone, USA) containing 10% fetal bovine serum, 100 U/mL penicillin, and 100 *μ*g/mL streptomycin at 37°C in 5% CO_2_.

### 2.3. CCK-8 Assay

After stable passage, SKOV3 and NuTu-19 cells were coincubated with increased concentrations of puerarin (0-320 *μ*g/mL) for 24 h and 48 h. Subsequently, the cells were seeded into a 96-well plate and CCK-8 solution (MedChemExpress, USA) was added into each well, followed by another 4 h of incubation. Then, the absorbance was detected using a microplate reader (CMaxPlus, MD) at 490 nm. Thereafter, appropriate concentrations of puerarin were used for subsequent experiments.

### 2.4. EdU Staining

After incubation with puerarin (0, 40, 80, and 160 *μ*g/mL) for 48 h, EdU staining was performed to assess OC cell proliferation according to the manufacturer's protocol. Firstly, 500 *μ*L of 2x EdU solution (Beyotime Biotechnology, China) was added into each well of the plate for 4 h of incubation; thereafter, 1 mL of 1x Hoechst 33342 solution (Beyotime Biotechnology, China) was added with further incubation for 2 min in the dark. Then, the cells were observed under a fluorescence microscope (Ts2-FC, Nikon, Japan).

### 2.5. Annexin V-FITC/PI Staining

Cell apoptosis assay was conducted by flow cytometry with the Annexin V-FITC/PI Apoptosis Detection Kit (BD Biosciences Pharmingen, USA) according to the standard protocol. Briefly, SKOV3 and NuTu-19 cells were treated the same as above. Then, the cells were harvested and washed with PBS and suspended in a binding buffer. After that, 5 *μ*L of Annexin V-FITC and 10 *μ*L of PI staining solution were added for 15 min of staining in the dark. Cell apoptosis was detected using flow cytometry (C6, BD Biosciences, USA).

### 2.6. Immunofluorescence Assay

The expression of PTEN and P53 in OC cells was detected using the immunofluorescence assay. After the cells were treated with puerarin, fixed with paraformaldehyde, permeated by 0.5% Triton X-100, and blocked with 3% BSA, the primary antibodies for PTEN and P53 were added for labeling at 1/500 dilution overnight at 4°C, followed by addition of the secondary antibody of goat anti-rabbit IgG H&L (Alexa Fluor® 488) for another 0.5 h of incubation. Then, DAPI staining solution (Servicebio, China) was used for nuclear staining. Finally, an inverted fluorescence microscope (Ts2-FC, Nikon, Japan) was used for image observation.

### 2.7. Animals

Female Fischer 344 rats (130–150 g) were purchased from Beijing Vital River Laboratory Animal Technology Co., Ltd. (Certification No. SCXK[Jing]2016-0006, China) and kept under specific pathogen-free conditions. After one week of adaptive feeding, rats were randomly divided into four groups with ten rats per group: normal control (NC) group, vehicle group, puerarin 1 group, and puerarin 2 group. The rats in the latter three groups received intraperitoneal injections of 1 mL of 5 × 10^6^/mL NuTu-19 cells. After cell injection, the rats in the puerarin 1 group received puerarin (solubilized in normal saline) at 40 mg/kg/d for nine weeks. The same dosage of puerarin was administered to the puerarin 2 group at the 7th week of cell injection for a total of three weeks. The rats in the NC group were instead injected with the same volume of normal saline after cell injection. All animal experimental procedures were approved by the Animal Experimentation Ethics Committee of Zhejiang Eyong Pharmaceutical Research and Development Center (Certification No. SYXK(Zhe)2021-0033).

### 2.8. Biological Property Detection and Sample Collection

During the experiment, the food intake, bodyweight, and survival outcome of the rats in each group were recorded. At the end of the experiment, the mice were euthanized by CO_2_ asphyxiation, and blood samples were collected and centrifugated to obtain the serum samples. Then, the abdominal cavity was opened, the lymph nodes in the abdominal cavity were photographed, and the ovaries were removed and weighed. The tumor nodes were removed; one part was fixed in 4% paraformaldehyde for histological and immunohistochemistry assays, while the rest was stored in liquid nitrogen for additional analysis.

### 2.9. Histological and Immunohistochemistry Analyses

The fixed OC tumor tissues were paraffin-embedded and cut into 5 *μ*m thick sections. Afterwards, the sections were stained with hematoxylin-eosin (HE, Servicebio, China), and the images were obtained with a microscope (NIKON DS-U3, Nikon). For TUNEL staining, after a series of pretreatment steps, the tumor tissue sections were stained with the TUNEL kit (Servicebio, China) and further stained with DAPI according to the manufacturer's instructions. The images were observed using a microscope. Immunohistochemistry analysis was performed to detect the positive expression of P53, PTEN, Ki67, and PCNA in tumor tissue sections using the corresponding antibodies.

### 2.10. Serum IL-6, TNF-*α*, and CA125 Detection

The levels of IL-6 and TNF-*α* in the serum of rats were detected using ELISA kits according to the standard protocol. The serum level of CA125 was detected using an electrochemiluminescence immunoassay with the relevant detection kit.

### 2.11. Flow Cytometry for T Cell Analysis

The peripheral blood samples from each group of rats were collected, stained on ice for lysing, and centrifuged. Then, the supernatant was removed, and PBS was added to the remaining sample to obtain a cell suspension. Subsequently, the cell suspension was incubated with the following antibodies: PE mouse anti-rat CD3, PE mouse anti-rat CD8a, and APC mouse anti-rat CD4. Next, T lymphocyte subsets were analyzed by C6 flow cytometry (BD Biosciences, USA).

### 2.12. Western Blot Assay

After the tumor tissues were homogenized on ice in the RIPA lysis buffer to collect the supernatant sample, the total protein concentration was detected using the BCA kit. Then, about 50 *μ*g of protein samples from each group was loaded onto 10% SDS-PAGE, followed by transfer onto nitrocellulose filter membranes. After blocking with nonfat milk, the membranes were incubated with the following primary antibodies: rabbit anti-rat P53, P21, PTEN, BRCA1, BIRC5, CTGF, Bax, cleaved caspase 3, and Bcl-2, followed by another 2 h of incubation with the secondary antibody. After that, the band images were visualized by ECL and quantified by ImageJ software.

### 2.13. 16S rDNA Sequencing and Bioinformatics Analysis of the Rat Gut Microbiota

The rat feces from each group were collected for gut microbiota analysis by 16S rDNA sequencing. Briefly, the DNA was extracted from rat feces samples from all groups using the E.Z.N.A.® Stool DNA Kit. Then, the DNA was electrophoresed using agarose gel and the DNA content was analyzed by an ultraviolet spectrophotometer according to the manufacturer's instructions. Next, amplification of the bacterial 16S rRNA gene was performed by PCR targeting the V3–V4 region using the specific primer: 341F (5′-CCTACGGGNGGCWGCAG-3′) and 805R (5′-GACTACHVGGGTATCTAATCC-3′). The amplicon library of the purified PCR products was quantified by the Qubit@ 2.0 Fluorometer (Thermo Scientific) using the Quant-iT PicoGreen dsDNA Assay Kit. Then, the library was sequenced on a NovaSeq platform at 2 × 250 bp paired-end reads. After that, clean reads were obtained from the raw data using the Cutadapt to remove the barcode and primer sequences. Low-quality sequences were filtered by Fqtrim, chimeras were filtered using vsearch (v2.3.4), and noise was reduced using DATA2 to finally obtain the feature sequences. Alpha diversity analysis, including Observed-species, Chao1, Shannon, Simpson, ACE, and Good-coverage, and beta diversity analysis on both weighted and unweighted UniFrac were performed by QIIME2 software and displayed with R software (v3.5.2). Species annotation was performed by sequence alignment using feature-classifier of QIIME2, and the reference databases were SILVA and NT-16S, with the SILVA database annotation results prevailing. Feature abundance was normalized using the relative abundance of each sample.

### 2.14. Statistical Analysis

SPSS 18.0 software was used to perform the statistical analysis, which included one-way ANOVA followed by the Tukey test. The data were presented as mean ± standard deviation (mean ± SD). Statistical significance was defined as *p* < 0.05.

## 3. Results

### 3.1. Puerarin Inhibits Cell Proliferation While Inducing Apoptosis of OC Cells

**Figure 1:**
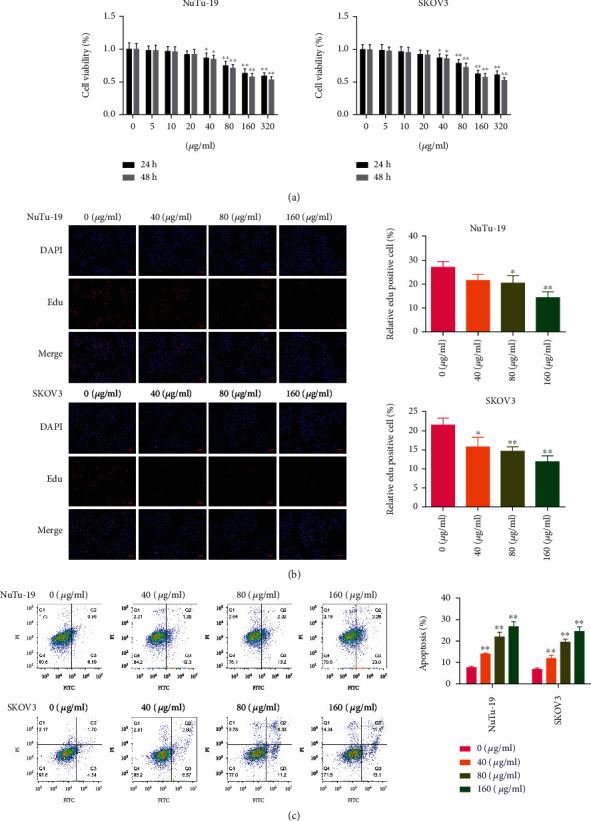
Puerarin inhibits OC cell proliferation and induces cell apoptosis. (a) OC cell lines NuTu-19 and SKOV3 were coincubated with increasing concentrations of puerarin (0-320 *μ*g/mL) for 24 h and 48 h, and CCK-8 assay was performed to detect the cell viability. (b) NuTu-19 and SKOV3 cells were coincubated with 0, 40, 80, and 160 *μ*g/mL of puerarin, and cell proliferation was detected using EdU staining. (c) Cell apoptosis rates of NuTu-19 and SKOV3 cells were assessed by Annexin V-FITC/PI staining with flow cytometry. The data are presented as mean ± SD, *n* = 3. ^∗^*p* < 0.05, ^∗∗^*p* < 0.01, compared to the 0 *μ*g/mL puerarin group. OC: ovarian cancer.

In SKOV3 and NuTu-19 cells incubated with puerarin at different concentrations, the cell viability decreased gradually, and statistical differences were observed at concentrations of 40–320 *μ*g/mL (Figure 1(a)). As there was no significant difference in the cell viability between the concentrations of 160 and 320 *μ*g/mL, concentrations of 40, 80, and 160 *μ*g/mL of puerarin were chosen for the subsequent experiments. EdU staining showed that EdU-positive cells were decreased in the puerarin-treated groups in both the SKOV3 and NuTu-19 cells (Figure 1(b)). Moreover, the cell apoptosis rates were significantly increased after puerarin treatment at all concentrations in the two cell lines (Figure 1(c)).

### 3.2. Puerarin Regulates the Expression of PTEN and P53 in OC Cells

**Figure 2:**
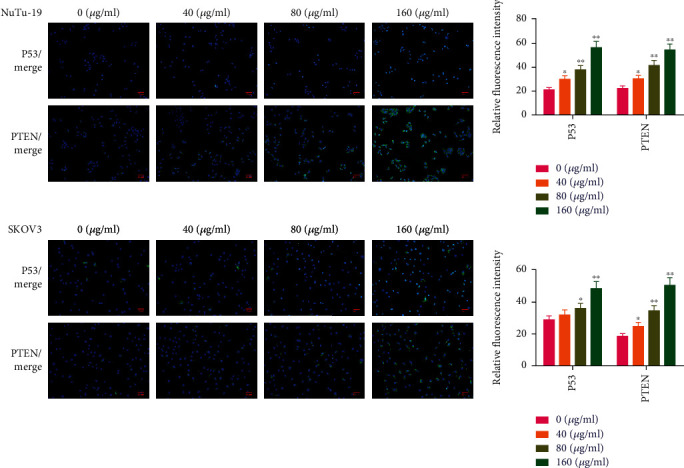
Puerarin activates the expression of P53 and PTEN in OC cell lines of NuTu-19 and SKOV3. The expression of P53 and PTEN in NuTu-19 and SKOV3 cells was detected by an immunofluorescence assay. The data are presented as mean ± SD, *n* = 3. ^∗^*p* < 0.05, ^∗∗^*p* < 0.01, compared to the 0 *μ*g/mL puerarin group. OC: ovarian cancer.

The immunofluorescence assay in Figure 2 shows that the expression levels of P53 and PTEN in untreated SKOV3 and NuTu-19 cells were very weak, while their expression was activated by puerarin in a dose-dependent manner, with the fluorescence intensities of P53 and PTEN gradually increasing in the cells of the puerarin-treated group.

### 3.3. Puerarin Suppresses Tumor Formation in OC Tumor-Bearing Fischer 344 Rats

**Figure 3:**
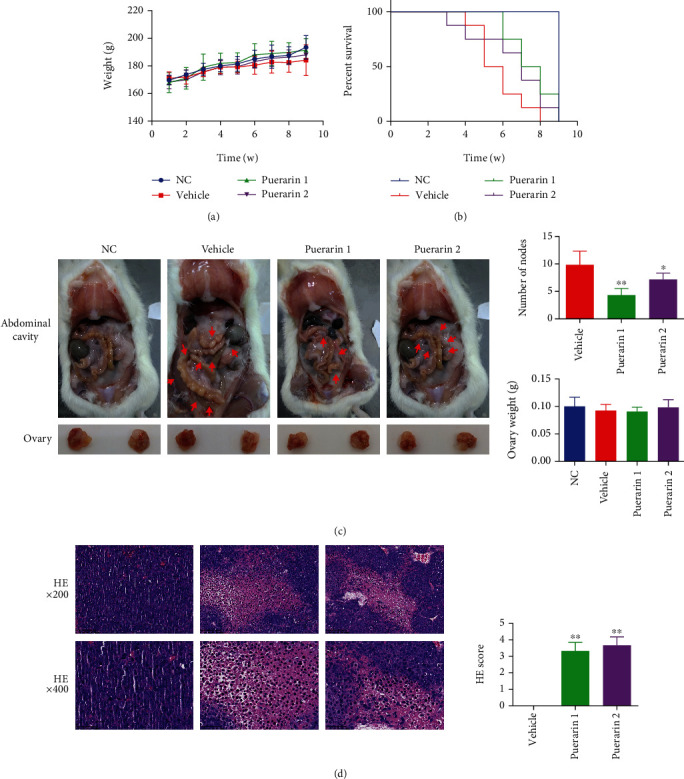
Puerarin inhibits ovarian tumor formation in Fischer 344 rats. (a) The change in bodyweight was recorded during the experiment. (b) The survival of the rats was recorded during the experiment. (c) The tumor nodes in the abdominal cavity of each group of rats were counted, and the ovarian weight was determined. (d) HE staining of the tumor tissues (magnification: ×200, 400). The data are presented as mean ± SD. ^∗^*p* < 0.05, ^∗∗^*p* < 0.01, compared to the vehicle group.

In the OC model rats, the bodyweight of the rats increased during puerarin administration, although there was no statistically significant difference among the groups (Figure 3(a)). The survival analysis of the rats in Figure 3(b) shows that there was no death event in the NC group, while all rats died by the 8th week in the vehicle group. After puerarin treatment, the survival time of the rats was prolonged in the puerarin 1 group, and they had a better survival outcome. In addition, obvious tumor nodes were observed in the abdominal cavity of the vehicle group rats, which were partly decreased in the puerarin treatment groups, with the least number of nodes in the puerarin 1 group (Figure 3(c)). While there were no statistically significant differences among the four groups regarding the ovarian weight, HE staining of the tumor tissues indicated that puerarin treatment led to different degrees of injury to the tumor tissues (Figure 3(d)).

### 3.4. Puerarin Promotes Cell Apoptosis and Regulates the Expression of Cell Apoptosis-Related Genes

**Figure 4:**
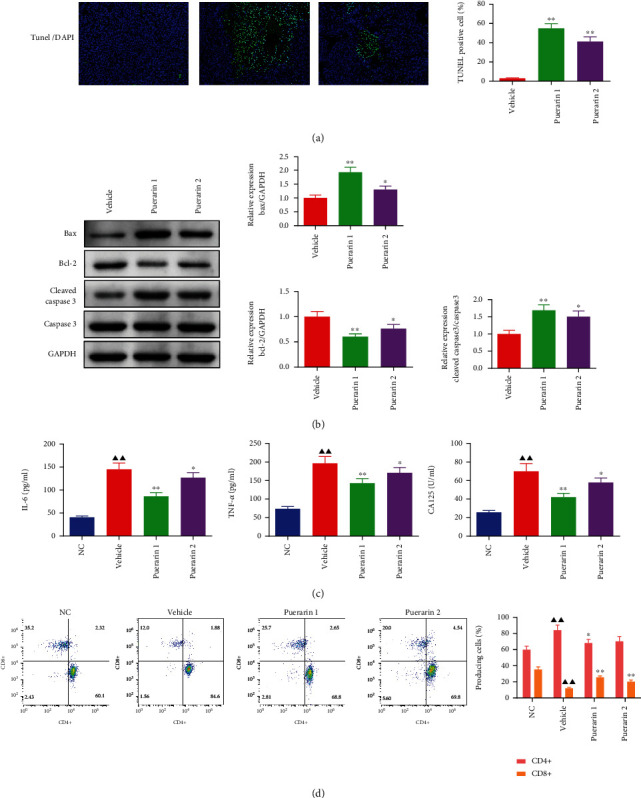
Puerarin treatment activates cell apoptosis and related proteins, while suppressing IL-6, TNF-*α*, and CA125 levels and modulating CD3+CD4+CD3+/CD8+ T lymphocyte percentages in OC tumor-bearing Fischer 344 rats. (a) TUNEL staining for the detection of cell apoptosis in OC tumor tissues (magnification: ×200). (b) Protein expression levels of Bax, Bcl-2, cleaved caspase 3, and caspase 3 in tumor tissues were detected by Western blot assay, and GAPDH was used as the internal control. (c) Serum levels of IL-6, TNF-*α*, and CA125 were detected by ELISA. (d) Flow cytometry was used to detect the CD3+CD4+CD3+/CD8+ T lymphocyte percentages. The data are presented as mean ± SD. ^▲▲^*p* < 0.01, compared to the NC group; ^∗^*p* < 0.05, ^∗∗^*p* < 0.01, compared to the vehicle group. OC: ovarian cancer.

TUNEL staining of the tumor tissues showed that TUNEL-positive cells in the tumor tissues were significantly increased in the puerarin 1 and puerarin 2 treatment groups (Figure 4(a)). Furthermore, compared to the vehicle group, the protein expression of Bax and cleaved caspase 3/caspase 3 was significantly increased, while that of Bcl-2 was significantly decreased in the tumor tissues after puerarin treatment (Figure 4(b)).

### 3.5. Puerarin Decreases the Serum Levels of IL-6, TNF-*α*, and CA125 and Modulates the Ratio of CD3+CD4+CD3+/CD8+ T Cells

It was observed that compared to the NC group, the serum levels of IL-6, TNF-*α*, and CA125 were significantly increased in the vehicle group, while puerarin treatment significantly inhibited the levels of these factors (Figure 4(c)). For T cells in the peripheral blood, the percentage of CD4+CD3+ T cells was increased, while that of CD8+CD3+ T cells was decreased in the vehicle group, and puerarin treatment reversed the percentage of CD4+CD3+/CD8+CD3+ T cells (Figure 4(d)).

### 3.6. Puerarin Regulates Cell Proliferation and the Expression of Anticancer-Related Genes

**Figure 5:**
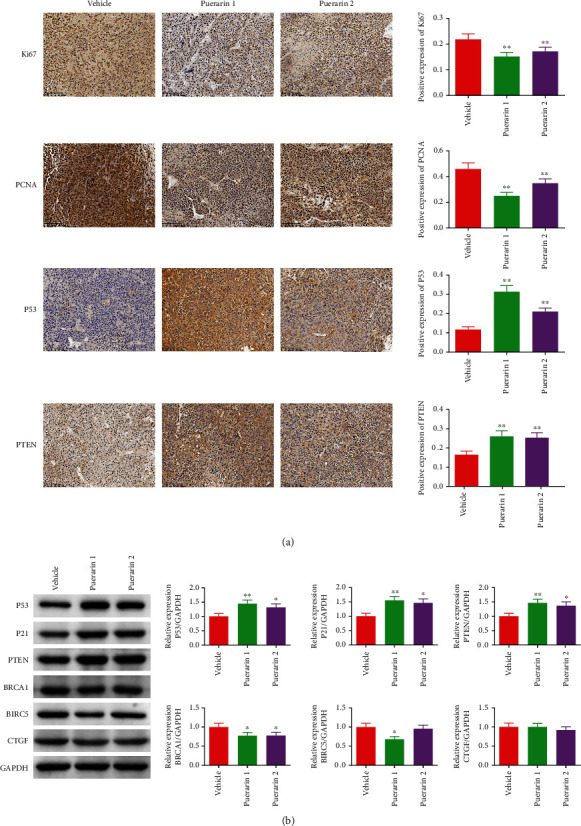
Expression of cell proliferation and tumor suppressor genes was regulated by puerarin treatment in OC model rats. (a) Positive expression of Ki67, PCNA, P53, and PTEN in tumor tissues was detected by an immunohistochemistry assay (magnification: ×200). (b) Protein expression levels of P53, P21, PTEN, BRCA1, BIRC5, and CTGF in tumor tissues were detected by Western blot assay, and GAPDH was used as the internal control. OC: ovarian cancer.

The expression of Ki67, PCNA, P53, and PTEN in tumor tissues was detected by an immunohistochemistry assay (Figure 5(a)). It was found that the expression levels of cell proliferation markers Ki67 and PCNA were significantly decreased in the puerarin-treated groups compared to the vehicle group, while the protein expression levels of P53 and PTEN were significantly increased in the puerarin-treated groups. Western blot results also indicated that the protein expression levels of P53, P21, and PTEN were increased, while those of BRCA1, BIRC5, and CTGF were decreased by puerarin treatment (Figure 5(b)).

### 3.7. Puerarin Modulates the Diversity of the Intestinal Microbes in OC Rats

**Figure 6:**
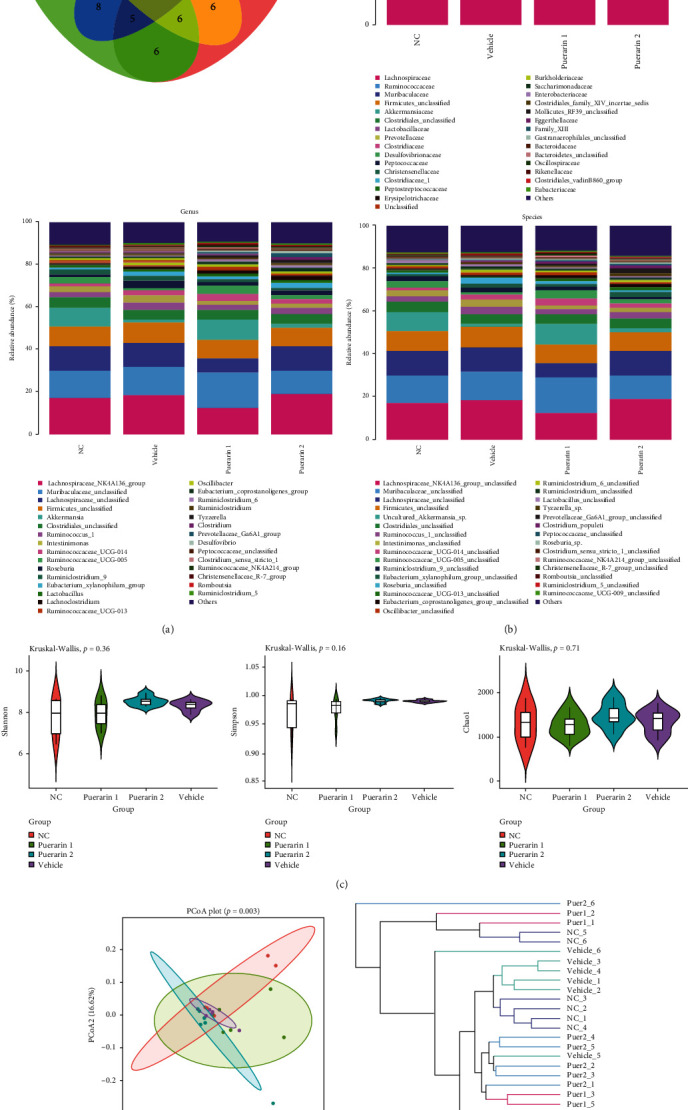
Puerarin modulates the diversity of the gut microbiota in OC rats. Rat feces were collected for 16S rDNA sequencing to analyze the effect of puerarin on the gut microbiota in OC model rats. (a) Venn diagram shows the features of the analyzed feces samples from all groups. (b) Stacked bar chart of the species richness analysis from the family, genus, to species of the detected microbes in the four groups. (c) Alpha diversity analysis, including the Shannon, Simpson, and Chao1 indexes, was used to assess the diversity of the intestinal microbes in the four groups. (d) Beta diversity of the intestinal microbiota was evaluated using principal coordinate analysis (PCoA) and Unweighted Pair Group Method with Arithmetic mean (UPGMA) clustering analysis. OC: ovarian cancer.

Rat feces were collected for 16S rDNA sequencing to analyze the effect of puerarin on the intestinal microbes in OC model rats. The Venn diagram in Figure 6(a) shows the similarities and differences among the rat feces samples of all groups. The stacked bar chart demonstrates species richness analysis from the family, genus, to species and shows that the abundance of the detected microbes varied among the four groups, suggesting that OC modeling and puerarin treatment altered the intestinal microbe composition in Fischer 344 rats (Figure 6(b)). Alpha diversity analysis, including the Shannon, Simpson, and Chao1 indexes, was used to assess the diversity of the intestinal microbes in the four groups. It showed that OC modeling and puerarin treatment changed the Shannon, Simpson, and Chao1 indexes (Figure 6(c)). In addition, the beta diversity of the intestinal microbiota was evaluated using principal coordinate analysis (PCoA). As shown in Figure 6(d), obvious separations among the groups were observed in S-plots of the PCoA (*p* = 0.003), indicating that the composition of the intestinal microbiota was changed in the four groups. The Unweighted Pair Group Method with Arithmetic mean (UPGMA) clustering analysis result is also presented in Figure 6(d).

### 3.8. Inhibitory Effect of Puerarin on OC Was Associated with Differences in the Gut Microbiota Communities among the OC Model Rats

**Figure 7:**
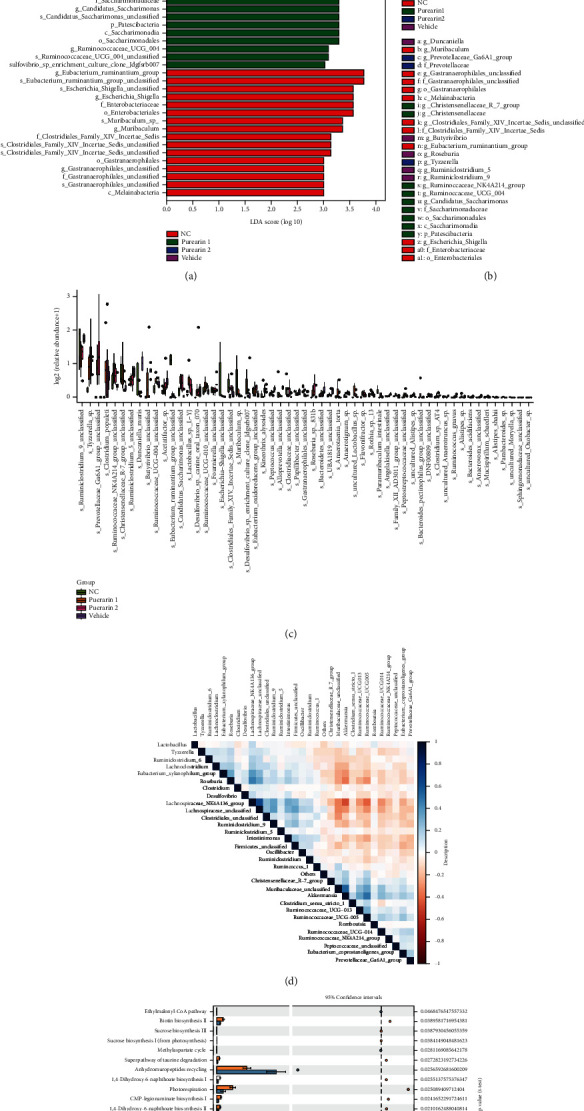
The inhibitory effect of puerarin on OC was associated with differences in the gut microbiota communities among the OC model rats. (a) Linear discriminant analysis Effect Size (LEfSe) analysis was performed to identify the key gut microbes. There were 49 markedly changed gut microbes with linear discriminant analysis (LDA) score > 3 and *p* < 0.05 among the four groups. (b) Cladogram of the identified gut microbes. (c) Expression of the significant gut microbes at the genus level in the four tested groups. (d) Correlation heatmap of the gut microbiota. (e) Metabolic pathway analysis of the gut microbes. OC: ovarian cancer.

To find the key biomarkers of the gut microbiota among the four groups, Linear discriminant analysis Effect Size (LEfSe) analysis was performed. The results showed that there were 49 markedly changed gut microbes with linear discriminant analysis (LDA) score > 3 and *p* < 0.05 among the four groups (Figure 7(a), Supplementary Table 1). These gut microbes mainly belonged to d__Bacteria.p__Bacteroidetes, d__Bacteria.p__Cyanobacteria, d__Bacteria.p__Firmicutes, d__Bacteria.p__Patescibacteria, etc. The cladogram in Figure 7(b) illustrates the phylogenetic relationships among the microbes in each group. Moreover, the expression of the significant gut microbes at the genus level in the four tested groups is shown in Figure 7(c). At the genus level, 44 kinds of significantly different gut microbes were observed, including g__Roseburia, g__Ruminiclostridium_9, g__Tyzzerella, g__Ruminococcaceae_NK4A214_group, and g__Christensenellaceae_R-7_group (*p* < 0.05). In addition, strongly positive (blue) and negative (red) correlations were observed among these microbiomes in the correlation heatmap (Figure 7(d)), suggesting that the gut microbiomes could interact with each other. In Figure 7(e), we analyzed the metabolic pathways of the gut microbes, which showed that these microbes were involved in multiple pathways, which affected the metabolism of the OC rats.

## 4. Discussion

OC is the most common cause of death among all cancers of the female reproductive system owing to its late presentation and highly malignant nature [1]. In recent years, researchers have made some progress in identifying new drugs and therapeutic strategies for OC treatment. In this regard, natural herbal products and their active compounds have been widely reported in the literature to have a favorable therapeutic effect [18]. In this study, we established *in vivo* and *in vitro* OC models to evaluate the anticancer activity of puerarin. We found that puerarin significantly inhibited cell viability and proliferation and induced cell apoptosis in OC cells. In OC model mice, tumor formation was suppressed, accompanied by the activation of tumor cell apoptosis and the expression of related proteins. The expression of tumor suppressor genes *P53* and *PTEN* was activated by puerarin in both OC cells and model rats. More importantly, the ratio of CD3+CD4+/CD3+CD8+ T cells recovered following puerarin treatment, and the results of 16S rDNA sequencing suggested that puerarin was involved in the modulation of the intestinal microbiome.

As a bioactive compound of herbal origin, puerarin is reported to have a wide range of pharmacological properties [15, 19, 20]. It also exerts protective action against various types of human cancers, and induction of cancer cell apoptosis is the main anticancer mechanism [16]. Tumor development is closely correlated with the inhibition of apoptosis or programmed cell death, which enables cancer cell immortality. Puerarin has been reported to have a chemopreventive effect on colon cancer HT-29 cells by reducing cell viability, inducing apoptosis, activating Bax and caspase 3, and inhibiting c-Myc and Bcl-2 [21]. Another study indicated that the induction of androgen-independent prostate cancer cell apoptosis by puerarin is associated with the suppression of the Keap1/Nrf2/ARE signaling pathway, and puerarin pretreatment resulted in an increase in Keap1 and decline in Nrf2, HO-1, and NQO1 protein expression [22]. In the present study, for investigating the anticancer effect of puerarin on OC, we also focused on the role of apoptosis. It was found that increasing concentrations of puerarin had an inhibitory effect on OC cell viability and proliferation in SKOV3 and NuTu-19 cells. Correspondingly, we also found that cell apoptosis was activated in OC cells, accompanied by an increased expression of Bax and cleaved caspase 3 and decreased expression of Bcl-2. Consistent with the results of previously reported studies, our findings also confirmed the anticancer effect of puerarin in OC cells and model rats. More importantly, it was shown that the induction of cell apoptosis as well as the regulation of related Bcl-2 and caspase family proteins contributed to the anticancer effect.

Furthermore, the elevated levels of serum IL-6, TNF-*α*, and OC-specific marker CA125 in OC rats were also decreased by puerarin administration. For cancer-elicited intrinsic inflammation, puerarin was able to regulate macrophage polarization and the expression of proinflammatory cytokines and genes, including TNF-*α*, IL-12, NF-*κ*B, and TLR4 [23]. Available studies have also suggested that puerarin is involved in the modulation of oxidative stress, cell cycle, and autophagy in cancer cells, which provides a new insight for further research on the role of puerarin in OC [24]. CA125 is the most widely used and important clinical biomarker for OC screening and prognosis [25–27]. The decreased level of CA125 in puerarin-treated groups suggests the cancer risk reduction potential of puerarin in OC.

During carcinogenesis, in contrast to oncogenes whose activation leads to the occurrence of cancer, tumor suppressor genes suppress the growth of cancer cells and contribute to the development of normal cells [28]. However, the function of these genes is often lost due to mutation, deletion, or inactivation in cancers. In OC cells and tumor tissues, we found that the expression of tumor suppressor genes *P53* and *PTEN* was activated in puerarin-treated groups, along with the altered expression of other related genes, including *P21*, *BRCA1*, *BIRC5*, and *CTGF*. This indicates that puerarin has the potential to activate the expression of tumor suppressor genes, thus contributing to the inhibition of OC cell survival and proliferation and induction of apoptosis. Mutations in *P53* are the most frequently occurring genetic event in OC, especially in high-grade OC, and accumulated evidence has revealed the significant role of *P53* in the diagnosis, progression, and prognosis of OC [29–31]. Puerarin is also reported to have the potential to mediate these genes and related pathways to protect from other diseases, including bone graft defects, cardiac toxicity, and hepatocellular carcinoma [32–35]. However, the clinical role of these genes remains controversial, and some studies have negated the clinical prognostic value of tumor suppressor genes and oncogenes in cancers [36]. Thus, we must realize that the “bench-to-bed” approach has a long way to go.

It is well accepted that the gut microbiome has a significant role in tumorigenesis and disease progression. Oncobiosis has been identified in numerous compartments, including the tumor tissue itself, female genital tract, serum, peritoneum, and intestines of OC patients [37]. Recent studies have also underlined a possible correlation between gut microbiota dysbiosis and OC carcinogenesis, treatment efficacy, and adverse effects [38]. In the present study, the results of 16S rDNA sequencing suggested that OC tumor modeling and puerarin treatment altered the gut microbiota diversity by varying degrees. A total of 49 types of significantly different gut microbes were identified in the analyzed groups that had close interaction with each other. It has been claimed that pathogenic bacteria, such as Gram-negative bacteria with high inflammatory potential, probably contribute to an increased risk of vaginal infections as well as the initiation and continuation of carcinogenesis [37]. In addition, the ratio of CD3+CD4+/CD3+CD8+ T lymphocytes was decreased by puerarin in OC rats in our study. CD3+, CD8+, and CD4+ T lymphocytes have a significant association with the level of tumor infiltration, which can also be related to the menopausal status, estrogen receptor (ER) status, Ki67 index, white blood cell (WBC) count, platelet count (PLT), lactate dehydrogenase (LDH), and CA153 in high-grade serous OC [39]. A study revealed that in all optimally debulked patients and in those undergoing paclitaxel/carboplatin therapy, the intraepithelial persistence of CD8+ T lymphocytes can improve the disease-free and overall survival of OC patients [40].

Furthermore, regarding puerarin administration, this study designed two groups with different drug administration start times; the puerarin 1 group was administered puerarin at the beginning of OC modeling and the puerarin 2 group after modeling. It was observed that the puerarin 1 group exhibited better antitumor effects. This indicates that the time point of drug treatment is also crucial for the therapeutic effect on the tumor. However, this study has some limitations. For investigating the antitumor effect of puerarin in OC, we mainly focused on the therapeutic mechanism of tumor suppressor genes and gut microbiota modulation. But the optimal approach and dose of puerarin that activates the expression of tumor suppressor genes remain unclear. Further in-depth studies are necessary to elucidate the exact role of puerarin in OC and explore new potential mechanisms systematically.

## 5. Conclusion

In conclusion, this study investigated the anticancer effect of puerarin in OC and found that puerarin treatment significantly inhibited OC cell viability and proliferation and induced cell apoptosis. In OC model mice, puerarin treatment suppressed tumor formation and modulated the gut microbiome. More importantly, the expression of tumor suppressor genes was activated by puerarin *in vitro* and *in vivo*. Hence, puerarin may be a potential agent for OC therapy.

## Data Availability

The data used to support the findings of this study are available from the corresponding author upon request.

## Supplementary Materials

Supplementary Materials Supplementary Table 1: 49 markedly changed gut microbes were identified by LEfSe analysis.
