# Supplementary material for: Anticancer Effect of Puerarin on Ovarian Cancer Progression Contributes to the Tumor Suppressor Gene Expression and Gut Microbiota Modulation
Source: J Immunol Res. 2022 Jul 28;2022:4472509. doi: 10.1155/2022/4472509 (PMC9352477; doi:10.1155/2022/4472509)
Supplement: Supplementary Materials — Supplementary Table 1: 49 markedly changed gut microbes were identified by LEfSe analysis. [file 4472509.f1.doc]

Supplementary table 1. 49 marked changed gut microbes were identified by LEfSe analysis

| Biomarker_names | Groups | LDA value | P value |
| --- | --- | --- | --- |
| d__Bacteria.p__Proteobacteria.c__Gammaproteobacteria.o__Enterobacteriales | NC | 3.57 | 0.005 |
| d__Bacteria.p__Firmicutes.c__Clostridia.o__Clostridiales.f__Lachnospiraceae.g__Butyrivibrio.s__Butyrivibrio_unclassified | Vehicle | 3.58 | 0.024 |
| d__Bacteria.p__Firmicutes.c__Clostridia.o__Clostridiales.f__Clostridiales_Family_XIV__Incertae_Sedis.g__Clostridiales_Family_XIV__Incertae_Sedis_unclassified.s__Clostridiales_Family_XIV__Incertae_Sedis_unclassified | NC | 3.14 | 0.024 |
| d__Bacteria.p__Firmicutes.c__Clostridia.o__Clostridiales.f__Lachnospiraceae.g__Tyzzerella.s__Tyzzerella_sp_ | Puerarin2 | 3.78 | 0.004 |
| d__Bacteria.p__Firmicutes.c__Clostridia.o__Clostridiales.f__Ruminococcaceae.g__Ruminococcaceae_UCG_004.s__Ruminococcaceae_UCG_004_unclassified | Puerarin1 | 3.09 | 0.020 |
| d__Bacteria.p__Firmicutes.c__Clostridia.o__Clostridiales.f__Ruminococcaceae.g__Ruminiclostridium_9 | Vehicle | 3.68 | 0.029 |
| d__Bacteria.p__Firmicutes.c__Clostridia.o__Clostridiales.f__Ruminococcaceae.g__Ruminococcaceae_NK4A214_group | Puerarin1 | 3.42 | 0.034 |
| d__Bacteria.p__Firmicutes.c__Clostridia.o__Clostridiales.f__Lachnospiraceae.g__Roseburia.s__Roseburia_sp__831b | Puerarin2 | 3.07 | 0.005 |
| d__Bacteria.p__Bacteroidetes.c__Bacteroidia.o__Bacteroidales.f__Muribaculaceae.g__Muribaculum | NC | 3.36 | 0.049 |
| d__Bacteria.p__Patescibacteria | Puerarin1 | 3.29 | 0.005 |
| d__Bacteria.p__Cyanobacteria.c__Melainabacteria.o__Gastranaerophilales.f__Gastranaerophilales_unclassified.g__Gastranaerophilales_unclassified.s__Gastranaerophilales_unclassified | NC | 3.01 | 0.004 |
| d__Bacteria.p__Firmicutes.c__Clostridia.o__Clostridiales.f__Lachnospiraceae.g__Butyrivibrio | Vehicle | 3.59 | 0.042 |
| d__Bacteria.p__Firmicutes.c__Clostridia.o__Clostridiales.f__Lachnospiraceae.g__Lachnoclostridium.s__Clostridium__populeti | Puerarin2 | 3.80 | 0.013 |
| d__Bacteria.p__Firmicutes.c__Clostridia.o__Clostridiales.f__Clostridiales_Family_XIV__Incertae_Sedis.g__Clostridiales_Family_XIV__Incertae_Sedis_unclassified | NC | 3.14 | 0.024 |
| d__Bacteria.p__Firmicutes.c__Bacilli.o__Lactobacillales.f__Lactobacillaceae.g__Lactobacillus.s__Lactobacillus_sp__L_YJ | Puerarin1 | 3.36 | 0.013 |
| d__Bacteria.p__Bacteroidetes.c__Bacteroidia.o__Bacteroidales.f__Muribaculaceae.g__Muribaculum.s__Muribaculum_sp_ | NC | 3.36 | 0.049 |
| d__Bacteria.p__Firmicutes.c__Clostridia.o__Clostridiales.f__Clostridiales_Family_XIV__Incertae_Sedis | NC | 3.14 | 0.024 |
| d__Bacteria.p__Patescibacteria.c__Saccharimonadia.o__Saccharimonadales | Puerarin1 | 3.29 | 0.005 |
| d__Bacteria.p__Bacteroidetes.c__Bacteroidia.o__Bacteroidales.f__Prevotellaceae.g__Prevotellaceae_Ga6A1_group.s__Prevotellaceae_Ga6A1_group_unclassified | Puerarin2 | 4.03 | 0.000 |
| d__Bacteria.p__Firmicutes.c__Clostridia.o__Clostridiales.f__Lachnospiraceae.g__Tyzzerella | Puerarin2 | 3.76 | 0.010 |
| d__Bacteria.p__Firmicutes.c__Clostridia.o__Clostridiales.f__Ruminococcaceae.g__Ruminococcaceae_NK4A214_group.s__Ruminococcaceae_NK4A214_group_unclassified | Puerarin1 | 3.42 | 0.034 |
| d__Bacteria.p__Bacteroidetes.c__Bacteroidia.o__Bacteroidales.f__Muribaculaceae.g__Duncaniella.s__Duncaniella_muris | Vehicle | 3.72 | 0.003 |
| d__Bacteria.p__Cyanobacteria.c__Melainabacteria | NC | 3.00 | 0.004 |
| d__Bacteria.p__Patescibacteria.c__Saccharimonadia.o__Saccharimonadales.f__Saccharimonadaceae.g__Candidatus_Saccharimonas.s__Candidatus_Saccharimonas_unclassified | Puerarin1 | 3.29 | 0.005 |
| d__Bacteria.p__Firmicutes.c__Clostridia.o__Clostridiales.f__Christensenellaceae.g__Christensenellaceae_R_7_group | Puerarin1 | 3.50 | 0.043 |
| d__Bacteria.p__Firmicutes.c__Clostridia.o__Clostridiales.f__Ruminococcaceae.g__Ruminococcaceae_UCG_004 | Puerarin1 | 3.09 | 0.020 |
| d__Bacteria.p__Cyanobacteria.c__Melainabacteria.o__Gastranaerophilales.f__Gastranaerophilales_unclassified | NC | 3.01 | 0.004 |
| d__Bacteria.p__Proteobacteria.c__Gammaproteobacteria.o__Enterobacteriales.f__Enterobacteriaceae.g__Escherichia_Shigella | NC | 3.57 | 0.014 |
| d__Bacteria.p__Cyanobacteria.c__Melainabacteria.o__Gastranaerophilales.f__Gastranaerophilales_unclassified.g__Gastranaerophilales_unclassified | NC | 3.01 | 0.004 |
| d__Bacteria.p__Patescibacteria.c__Saccharimonadia | Puerarin1 | 3.29 | 0.005 |
| d__Bacteria.p__Proteobacteria.c__Gammaproteobacteria.o__Enterobacteriales.f__Enterobacteriaceae.g__Escherichia_Shigella.s__Escherichia_Shigella_unclassified | NC | 3.57 | 0.014 |
| d__Bacteria.p__Firmicutes.c__Clostridia.o__Clostridiales.f__Christensenellaceae | Puerarin1 | 3.51 | 0.046 |
| d__Bacteria.p__Cyanobacteria.c__Melainabacteria.o__Gastranaerophilales | NC | 3.01 | 0.004 |
| d__Bacteria.p__Firmicutes.c__Clostridia.o__Clostridiales.f__Christensenellaceae.g__Christensenellaceae_R_7_group.s__Christensenellaceae_R_7_group_unclassified | Puerarin1 | 3.50 | 0.043 |
| d__Bacteria.p__Firmicutes.c__Clostridia.o__Clostridiales.f__Lachnospiraceae.g__Roseburia | Vehicle | 4.13 | 0.027 |
| d__Bacteria.p__Proteobacteria.c__Gammaproteobacteria.o__Enterobacteriales.f__Enterobacteriaceae | NC | 3.57 | 0.005 |
| d__Bacteria.p__Bacteroidetes.c__Bacteroidia.o__Bacteroidales.f__Prevotellaceae | Puerarin2 | 4.03 | 0.018 |
| d__Bacteria.p__Patescibacteria.c__Saccharimonadia.o__Saccharimonadales.f__Saccharimonadaceae.g__Candidatus_Saccharimonas | Puerarin1 | 3.29 | 0.005 |
| d__Bacteria.p__Firmicutes.c__Clostridia.o__Clostridiales.f__Ruminococcaceae.g__Ruminiclostridium_9.s__Ruminiclostridium_9_unclassified | Vehicle | 3.68 | 0.029 |
| d__Bacteria.p__Firmicutes.c__Clostridia.o__Clostridiales.f__Lachnospiraceae.g__Acetatifactor.s__Acetatifactor_sp_ | Vehicle | 3.13 | 0.046 |
| d__Bacteria.p__Patescibacteria.c__Saccharimonadia.o__Saccharimonadales.f__Saccharimonadaceae | Puerarin1 | 3.29 | 0.005 |
| d__Bacteria.p__Proteobacteria.c__Deltaproteobacteria.o__Desulfovibrionales.f__Desulfovibrionaceae.g__Desulfovibrio.s__Desulfovibrio_sp__canine_oral_taxon_070 | Puerarin2 | 3.71 | 0.012 |
| d__Bacteria.p__Firmicutes.c__Clostridia.o__Clostridiales.f__Lachnospiraceae.g__Eubacterium__ruminantium_group.s__Eubacterium__ruminantium_group_unclassified | NC | 3.77 | 0.006 |
| d__Bacteria.p__Firmicutes.c__Clostridia.o__Clostridiales.f__Lachnospiraceae.g__Eubacterium__ruminantium_group | NC | 3.77 | 0.006 |
| d__Bacteria.p__Proteobacteria.c__Deltaproteobacteria.o__Desulfovibrionales.f__Desulfovibrionaceae.g__Desulfovibrio.s__Desulfovibrio_sp__enrichment_culture_clone_Jdgsrb007 | Puerarin1 | 3.03 | 0.008 |
| d__Bacteria.p__Bacteroidetes.c__Bacteroidia.o__Bacteroidales.f__Muribaculaceae.g__Duncaniella | Vehicle | 3.72 | 0.003 |
| d__Bacteria.p__Bacteroidetes.c__Bacteroidia.o__Bacteroidales.f__Prevotellaceae.g__Prevotellaceae_Ga6A1_group | Puerarin2 | 4.03 | 0.000 |
| d__Bacteria.p__Firmicutes.c__Clostridia.o__Clostridiales.f__Ruminococcaceae.g__Ruminiclostridium_5.s__Ruminiclostridium_5_unclassified | Vehicle | 3.24 | 0.031 |
| d__Bacteria.p__Firmicutes.c__Clostridia.o__Clostridiales.f__Ruminococcaceae.g__Ruminiclostridium_5 | Vehicle | 3.24 | 0.031 |
